# Remote Delivery of Motor Function Assessment and Training for Clinical Trials in Neuromuscular Disease: A Response to the COVID-19 Global Pandemic

**DOI:** 10.3389/fgene.2021.735538

**Published:** 2021-10-29

**Authors:** Meredith K. James, Kristy Rose, Lindsay N. Alfano, Natalie F. Reash, Michelle Eagle, Linda P. Lowes

**Affiliations:** ^1^ The John Walton Muscular Dystrophy Research Centre, Newcastle University and Newcastle Hospitals NHS Foundation Trust, Newcastle Upon Tyne, United Kingdom; ^2^ Discipline of Physiotherapy, Faculty of Medicine and Health, University of Sydney, Sydney, NSW, Australia; ^3^ Sydney Children's Hospitals Network, Sydney, NSW, Australia; ^4^ Center for Gene Therapy, The Abigail Wexner Research Institute at Nationwide Children’s Hospital, Columbus, OH, United States; ^5^ Department of Pediatrics, The Ohio State University, Columbus, OH, United States; ^6^ ATOM International, Newcastle Upon Tyne, United Kingdom

**Keywords:** COVID-19, natural history, clinical outcome assessment (COA), neuromuscular disorders (NMD), physical therapy, telemedicine, clinical trials

## Abstract

Clinical outcome assessments of function or strength, assessed by physical therapists, are commonly used as primary endpoints in clinical trials, natural history studies and within clinics for individuals with neuromuscular disorders. These evaluations not only inform the efficacy of investigational agents in clinical trials, but also importantly track disease trajectory to prospectively advise need for equipment, home and work modifications, and other assistive devices. The COVID-19 pandemic had a global impact on the safety and feasibility of in-person visits and assessments, necessitating rapid development of mitigation strategies to ensure ongoing collection of key clinical trial endpoints and access to expert clinical care despite travel restrictions. Physical therapists who are expert in neuromuscular disorders working across clinics, countries, and clinical trials developed initial guidelines and methods for the suitability and feasibility of performing remote evaluations. A number of Sponsors introduced amendments to their study protocols to enable remote evaluations, supported by live video streaming of the assessment to their local clinical evaluators. Similarly, application of these techniques to clinical telemedicine enabled objective evaluations for use in payer discussions, equipment procurement, and general access to expert physical therapy services. Here we report on our methodology for adapting current practices to remote testing and considerations for remote evaluations.

## Introduction

The COVID-19 pandemic presented unprecedented challenges to the delivery of clinical care and the conduct of clinical outcome assessments (COA) for individuals with neuromuscular disorders (NMD). Due to the frequent comorbidities seen in NMD, it was considered a significant risk for many patients to leave their homes and attend hospital clinics during the height of the pandemic and subsequent waves. This was further exacerbated by heavily restricted travel within and between states, and countries globally. To ensure the continuation of clinical care and clinical trials, COVID-19 mitigation strategies were urgently required to accommodate circumstances where patients were unable to travel to their site.

Prior to the COVID-19 pandemic, remote testing and telehealth assessment were not routinely part of standard clinical practice and less often part of clinical trials in NMD. Complicating matters further, the remote administration of existing COAs had not been validated in any patient population with NMD. However, in the midst of the pandemic, replacement of in person visits with video consultations and utilizing telemedicine was considered to be the most appropriate option for end point collection of clinical trial primary and secondary COA normally performed by Physical therapists (PTs).

National regulatory agencies including the United States Food and Drug Administration (FDA, U.S. Department of Health and Human Services, 2020), European Medicines Agency (EMA, Europen Medicines Agency, 2020), the Medicines and Healthcare products Regulatory Agency (MHRA, Medicines and Healthcare products Regulatory Agency, 2020) and National Health Service in the UK (NHS, Health Research Agency, 2020) issued guidance on the management of clinical trials during the pandemic. Given the majority of the NMD population were advised to shield, there was an urgent need to minimize risk to study participants. For NMD trials already underway, the guidance allowed, with discussion and approvals from with relevant agencies, rapid adaptations to protocols and procedural requirements. This included reduced travel to hospital sites for study visits and delaying face to face visits. For most studies, flexibility was given for consent methods for COVID-19 related protocol amendments, including being able to consent over the telephone or via video call. Additionally, some investigational products could be delivered and administered at home via visiting nursing staff.

Within local and national health services, rapid adoption of approved telemedicine platforms facilitated video and telephone clinics (Triki et al., 2021), removing need for many patients to attend face to face clinics whilst shielding. Standard of care assessments normally completed in the clinic required review and adaptation to ensure a patient’s progress continued to be monitored. A further complication was the redeployment or furlough of PTs, necessitating the training of new staff. In order to ensure the new staff continued to receive a similar standard of training to that provided pre-pandemic, training methodology also needed to be modified for remote delivery.

In this paper, we elaborate on the feasibility of adapting clinic-based COAs to remote-based evaluations for both research and clinical applications. We discuss important methodological considerations for these adaptations and present our experiences, both positive and negative.

## Methods

Specialized neuromuscular (PTs) with expertise in clinical trial design and outcome measures met remotely to explore the feasibility of adapting COA typically conducted in the clinic setting for remote administration. Our goal was to both facilitate continuity of care to clinic patients and maintain the capacity to collect critical clinical trial endpoints. Essential considerations for success of any remote testing protocol and to enable their validation, were to maintain patient safety while also providing standardized assessments that closely mimicked clinical practice and environments. Initial guidelines for the suitability and feasibility of performing remote evaluations of COA commonly used in clinical trials were developed. This initiative was supported by a number of study Sponsors, who were in turn supported by national regulatory bodies enabling rapidly amended protocols for remote evaluations to occur. In conjunction with Sponsors, expert PTs reviewed study protocols and schedule of events to determine the suitability and feasibility of all study COA that were normally collected by PTs in the clinic or trained Clinical Evaluators (CEs) for clinical trials, for remote evaluation, where the PT administered instructions via video call.

### Locations for Remote Evaluations

Three main alternatives were considered suitable for patients to be evaluated remotely during the COVID-19 pandemic. The option chosen was largely dependent on family preference, local travel restrictions and regulatory approvals in place.

1 The patient was evaluated in their home with a caregiver present under the direction of the PT/CE from the patient’s clinic or study site. The caregiver acted as an assistant and was available to facilitate, conduct, or be available for safety (as needed) by the PT/CE via video conferencing.
2 The patient was evaluated at a facility close to their home by their local PT under the guidance of the CE from the patients’ study site. The local PT acted as the facilitator and completed assessments under the guidance of the CE via video conferencing.
3 The patient was evaluated in their home by the local PT/study CE who travelled to the home if this was deemed safe and acceptable by the patient, Sponsor, and regulatory authorities.

While most evaluations were completed according to the first option, the others were offered for flexibility and to adapt to each patient and family’s unique needs and level of comfort with telehealth methods.

### Evaluation of the Suitability of Existing COAs for Remote Administration

**FIGURE 1:**
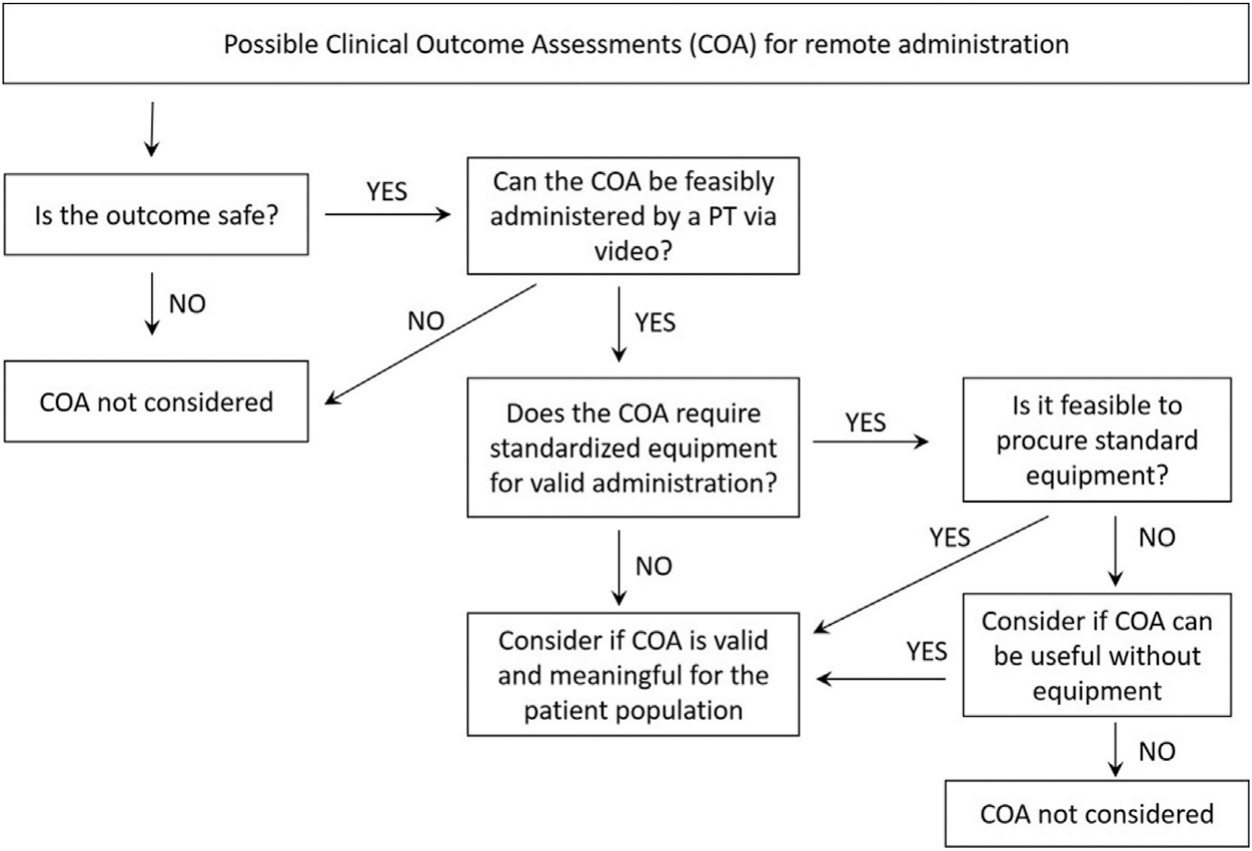
Algorithm used to guide decision making regarding the suitability of COA for remote administration.

The first step in selection of appropriate remote COA was to carefully evaluate each assessment (Figure 1) to ensure the assessment could be 1) safely administered in the home with instructions given by PT/CE via video feed 2) performed in the same standardized manner as in clinic visits to enable comparison of data across environments, 3) accurately and reliably scored over a video feed 4) completed so that data be collected and transmitted to site in a confidential manner.

#### Safety Considerations: Impact on the Selection of COAs for Remote Administration

To ensure safety during the remote evaluation, each COA was reviewed for the potential risk for both patient and caregiver when administered remotely. Clinically, the patient is often asked to attempt difficult items to determine their highest abilities, although this is under the supervision of a skilled PT. Items such as stair climbing, stepping up on elevated surfaces, and other balance activities can lead to falls which potentially can cause significant injury. A skilled PT can minimize the risk of injury to the subject as well as themselves through careful, ergonomically correct guarding techniques. It was recognized that caregivers may not know these techniques, which could put themselves and the patient at risk for injury. Some assessments, such as the 4-stair climb were removed from consideration because of safety concerns. COA that required a high level of patient handling from a skilled PT in facilitating movement or required an environment such as an internal 30-m corridor were not deemed suitable. Those such as quantitative muscle strength testing, Children’s Hospital of Philadelphia Infant Test of Neuromuscular Disorders (CHOP Intend) (Glanzman et al., 2010) and the 6 min walk test (6MWT) (Enright et al., 2003), 100 m timed test (Alfano et al., 2017), among others.

Following the safety review, the expert group had identified COAs or individual items of assessments that were potentially feasible to complete via video assessment in the home Table 1 shows the NMD and COA considered feasible for remote administration. Those COA marked with an Asterix indicates those thought to require modification for remote assessment. With sufficient preparation and equipment, many of the assessments could be completed. Standard operating procedures and PT manuals were developed for remote administration.

**TABLE 1:** Pediatric and adult patient populations and outcomes tested remotely across expert neuromuscular centers in response to the COVID-19 pandemic.

Patient populations			
Clinic		Research	
−SMA	−LAMA2	−DMD	
−LGMD	−FSHD	−LGMD	
−DMD/BMD	−Myotonic dystrophies	−SMARD1	
−CMD	−CMS	−SMA	
−Congenital myopathies	−VCP	−VCP	
−HMERF	−CMT		
−Pompe disease	−Myofibrillar myopathies		

**Clinical Outcome Assessments**			
**Clinic**		**Research**	
−Patient reported outcomes	−RHS/HFMSE	−NSAA	−9HPT
−NSAA/NSAD	−CHOP Intend^*^	−NSAD	− Box and Blocks
−Neuromuscular GRO	−RULM ^	−TUG	− RHS/HFMSE
−Bayley	−EK2	−PUL	− Bayley (gross motor)
−MFM* ^	−Brooke/PUL entry	−Brooke	− Motor milestones
−TFT ^	−Vignos	−Neuromuscular GRO	− Spirometry
		−TFT	− CHOP Intend^*^

* Indicates the assessment was attempted but needed to be modified or performed partially when conducted remotely due to safety and/or handling expertise required. ^ Indicates the assessment can be feasible with standardized equipment and/or sufficient space within home which may not be available for some visits.

### Guidelines to Ensure the Standardization of COA Administration

#### Standardization of Equipment Across Settings

To maximize the validity of the remote assessment, standardisation of administration was crucial. It was a priority the equipment and home environment mirrored the clinic setting as closely as possible. This was not only important to produce valid and reliable data but to compare scores longitudinally. In PT departments a variety of standardised equipment is available which is not always the case in the family home.

Where funding was available standardized equipment including adjustable height benches, aerobic steps, and Performance of Upper Limb (Mayhew et al., 2020) kits were procured for remote use and shipped to the family. Logistical planning was required for all necessary equipment to arrive at a family’s home prior to any remote testing. Pandemic related equipment shortages and prolonged shipping time were taken into consideration when scheduling patient visits. Common sense and clinical sensibility were considered to determine which equipment was essential to complete key endpoints to reduce the burden of ongoing study participation for the family. For example, large sets of 4 standard stairs was not deemed reasonable to send to a family home compared to a small aerobic step.

#### Standardization of the home testing environment

It was important to ensure the home environment mimicked the clinic as much and as safely as possible. Adequate space and consistent flooring such as low pile carpeting or tile, linoleum, or hardwood was a priority. Any outdoor surfaces were deemed unsuitable due to the same safety concerns and difficulty in standardization.

#### Accuracy and Reliability of Scoring Via Live Feed

While initially satisfied all COAs selected could be reliably scored via video or live feed, early in the implementation of remote evaluations we became concerned about the accuracy of timing assessments via the live video stream. This was particularly apparent when a poor internet connection resulted in buffering. To test this hypothesis, we conducted a series of timed tests with one physical therapist timing in the home and one timing remotely via the web feed. We found the traditional protocol of starting the watch when the examiner said ‘go’ added about a second to the actual performance time. We attributed this to the time required for the sound to travel to the subject and the video movement to return to the therapist. To eliminate this discrepancy, we instead instructed the caregiver at home to say ‘go’. This technique provided consistently reliable results with an acceptable tolerance ± 0.3 s between the in-home therapist and web-based therapist. For some clinical trials, evaluations were required to be videoed. Where a video of the timed test was recorded by the caregiver during the live evaluation, the test could be timed via the video. Using this method allowed timing from when the command ‘Go’ from the PT was heard in the home, and also negated issues over internet connection speed.

#### Data Could Be Collected and Transmitted to the Site Via Procedures Compliant with Local Data Security, Privacy Regulations and Laws

A compliant webservice, for collecting and transmitting the data approved by national or state health service providers, clinical trial regulators and local Ethical approval boards was utilized for all occasions of remote assessment.

### Practice Guidelines Utilized for the Conduct of Remote Assessments

Best practice guidelines for the remote conduct of COAs were developed by the expert PT group. This was needed to satisfy local laws and regulations (Tucker et al., 2016), particularly with regard to data security and privacy and to ensure evaluations were conducted safely and in a highly reliable and standardized manner.

#### Accurate Patient Identification and Privacy Considerations

Staff were required to document how services were provided (I.e. document video-conference platform), confirm patient identity (I.e. date of birth, visual recognition, etc), the patient and family location at the time of the visit, and the provider’s location. The session was conducted in a private location so that other individuals could not observe screens or overhear a conversation. The families were instructed to use a secure password-protected Wi-Fi network rather than a public connection. The device and platform used to conduct the visit was documented.

#### Establish and Document Patient/Family Consent

As with any protocol amendment or provision of clinical care, it was essential to obtain patient and/or parent/guardian consent and assent in accordance with a clinic’s local, state, and federal practices. Specifically, consent was required for remote videography, if applicable, furthermore, caregivers were instructed not record others in the home. Siblings, pets and other distractions were instructed to be moved to another part of the house to ensure a quiet, uninterrupted space for testing. The view captured on the recording was checked for appropriate view of the patient.

#### Conducting Valid Remote Assessments

Prior to the first formal remote assessment a pre-evaluation call with the patient and family was required to ensure all equipment was received and in good working order, to identify suitable testing spaces, and familiarize all parties with the process and procedures. This practice call provided study staff with an opportunity to test the home internet bandwidth, video-conferencing technology, and identify and troubleshoot any issues. The technology check was conducted in the locations the remote assessment would take place, on the digital devices to be used for the assessment.

The practice run included all the elements of the formal assessment, ensuring appropriate environment, patient clothing, floor surfaces, testing audio for delivery of instructions from the clinical evaluator, camera location and a practice of the relevant tests, particularly the timed tests with video capture. With particular relevance for the timed tests, it was important the parent video-recording from the practice session could be reviewed live by the CE to ensure that an accurate assessment of the test could be collected from the video, including ensuring that the patient was in view and that the CE commands heard. The practice also ensured a harmonisation of the language typically used to conduct the assessment to the home environment. There were instances where patients were visiting sites from other countries and did not speak the local language of the CE. Simultaneous translation via a separate line ensured engagement and understanding of the patient and caregiver.

To remain consistent with standard practice, evaluation of COA were recommended to be performed in the morning to capture the patient’s abilities before they fatigue across the day. Scheduling remote visits required some flexibility from the CE if the patient resided in a different time zone. The patient’s time zone was considered priority when scheduling a visit.

It was recommended the same CE complete evaluations with the patient to reduce any variability in measurement and online interaction. Similarly, to reduce other sources of error, all remote evaluations were performed in the same physical location in the home, at the same time of day, and preferably with the same CE and parent/caregiver combination. Having two caregivers present in the home was helpful to ensure one caregiver would fully engage with the patient with a focus on safety, while the second managed the technology and ensure correct video with the patient remaining within the camera view. Additionally, to optimize the video feed, we recommended a tripod be used so the camera could be kept in one place. A caregiver/patient instruction manual was provided to explain the remote video evaluation process, the assessments and required equipment.

To safeguard data quality and consistency CEs were instructed to document unsafe items as ‘not attempted’ rather than score the person as ‘unable to complete’ the item, as this would artificially and invalidly lower a subject’s score.

## Discussion

### The Feasibility of the Proposed Remote Testing Model

During this experience we have observed success with remote evaluations for both clinical and research applications. Our teams have jointly evaluated 300 + patients in clinical practice and in research trials across 225 + visits. Remote visits were introduced to seven research trials, in 20 trials sites across the United Kingdom, United States, Australia and Europe. Clinical work was completed at three specialist NMD centres in the UK, USA and Australia. Table 1 summarized our work to date and demonstrates the variety of patient populations and outcomes assessed via telehealth and video conferencing methods. Table 2 is a matrix of NMD conditions and possible COA options. In our collective experience, we have reached >90% of patients to ensure ongoing access to clinical care and ongoing collection of key trial endpoints. The results of the remote evaluations are not included in this paper. Early on in the pandemic, telehealth was required for patient and clinician safety. However, as governments and hospitals in some regions are beginning to relax restrictions, some families are continuing to choose telehealth options for their ongoing clinical care. In the interim, research trials are also providing some flexibility for visit type to promote ongoing trial participation and maximize patient safety.

**TABLE 2:** Matrix of patient populations and outcomes tested remotely. Refer to Table 1 for overview of which outcomes were administered clinically or for research purposes. Outcomes that require standardized equipment were typically only completed for research purposes where equipment could be supplied. For clinical purposes, modified versions of some scales ( ^ ) may have been attempted remotely if required for treatment authorizations in some patient cohorts and regions. 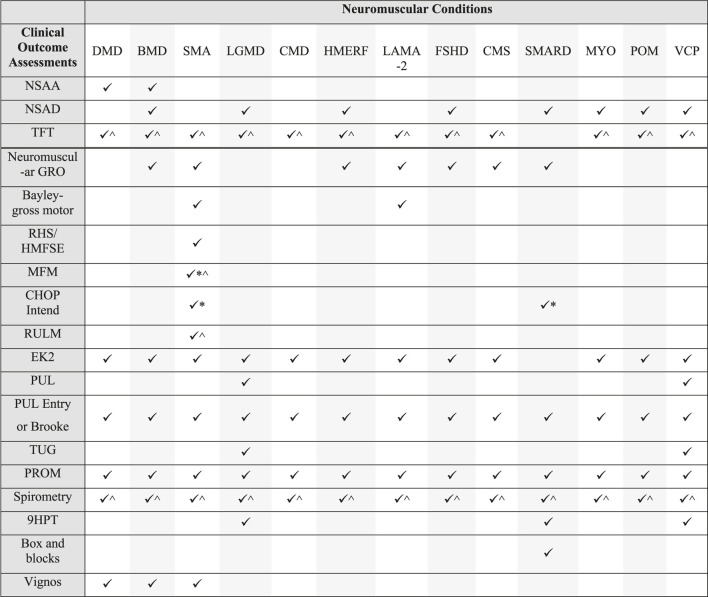

* Indicates the assessment was attempted but needed to be modified or performed partially when conducted remotely due to safety and/or handling expertise required. ^ Indicates the assessment can be feasible with standardized equipment and/or sufficient space within home which may not be available for some visits. DMD: Duchenne muscular dystrophy; BMD: Becker muscular dystrophy; SMA: spinal muscular atrophy; LGMD: limb girdle muscular dystrophy; CMD: Congenital muscular dystrophies; HMERF: Hereditary myopathy with early respiratory failure; LAMA2: laminin-2 related muscular dystrophy; FSHD: facioscapulohumeral dystrophy; CMS: congenital myasthenic syndromes; SMARD1: SMA with respiratory distress; POM: Pompe disease; MYO: Myotonic dystrophy; VCP: valosin containing protein associated multisystem proteinopathy; NSAA: North Star Ambulatory Assessment; NSAD: North Star Assessment for limb girdle-type dystrophies; TFT: Timed functional tests including rise from floor, 10 m walk/run; Neuromuscular GRO: Neuromuscular Gross Motor Outcome; Bayley: Bayley scales of infant and toddler development–gross motor subtest; MFM: Motor Function Measure; RHS: Revised Hammersmith Scale; HFMSE: Hammersmith Functional Motor Scales Expanded; CHOP Intend: Children’s Hospital of Philadelphia Infant Test of Neuromuscular Disorders; RULM: Revised Upper Limb Module; EK2: Egen Klassifikation two; Brooke: Brooke Upper Extremity Functional Rating Scale; PUL: Performance of Upper Limb; Vignos: Vignos Lower Extremity Rating Scale; TUG: Timed Up and Go; PROM: Patient report outcome measure; 9HPT: Nine hole peg test.

### Benefits of a Remote Option for Healthcare Provision and COAs

There are many benefits to providing clinical assessment and care to a patient and their family within the home environment. The PT has the opportunity to assess the family unit within their natural environment and can gain extra context around strengths and issues specific to the patient’s home. Problem solving and remedies can be readily suggested using items or equipment already existing within the home or suggest more specific adaptations based on unique furniture or home floorplans.

From a research perspective, testing a patient within their home environment has the potential to add ‘real-world’ validity and clinical meaningfulness to the evaluation. Results are directly applicable to what a patient can actually do within their home. When assessing items on the Performance of Upper Limb, for example, tabletop activities performed on the family dining room table can be correlated to the patient’s independence with eating, drinking, and other tabletop tasks. For paediatric and adult patients, there is also the benefit of feeling more comfortable, safe, and at ease within the home environment.

Remote testing and telehealth decentralize testing and care and reduces the many burdens associated with travel to a local, national, or international centre of excellence for standardized testing. Travel itself is fatiguing, stressful and can impact a patient’s consistency and accuracy with evaluation. Decisions about clinical care or efficacy of clinical trials using these data can be flawed if the fatigue of travel washes out potential treatment effects. Similarly, other stressors such as missed work, school, and financial burden associated with this travel are reduced when care and visits can be completed within a preferred environment.

In rare and ultrarare patient populations, remote testing has the potential to vastly expand potential recruitment pools reducing barriers to trial participation such those mentioned above. Completing visits and evaluations within a home environment could lead to increased recruitment potential and generalizability of study results.

### Limitations and Considerations for Implementation of Remote Evaluations

The two largest challenges to date have been sufficient internet/broadband access and patient/caregiver comfort with technology. While we have had abundant success with providing care and completing evaluations remotely, there have been some instances where internet access is insufficient to have a conversation with a patient/caregiver or complete a standard evaluation via live video feed. Mitigation of these issues can occur but differ across clinical and research applications. For example, in a clinical setting where video feeds are limiting, it is possible to provide some level of care via phone call. In other situations where the internet disturbance is deemed to be temporary, a follow up video visit has been scheduled on a different date.

Individuals have a spectrum of comfort and competency with technology. A small number of caregivers or patients decline to participate in remote evaluations due to lack of familiarity with technology, which unfortunately resulted in a lack of data until the given patient could safely return onsite for assessments. However, most patients and caregivers were willing to attempt remote assessments. We offered reassurance that it was a learning process for all involved and the practise sessions were helpful in mitigating concerns. or technical issues before the actual data collection assessment.

Evaluations conducted in the clinic allow for a consistent PT to position, instruct and motivate the subject. When clinical trial evaluations take place in the clinic setting, they almost always prohibit the caregiver from participating, to ensure a consistent testing dynamic. However, restricting caregiver access to the visit within the home environment is simply not feasible or recommended. While there can be many positives to inclusion of parents and caregivers in a testing visit, it is essential that data privacy be maintained for research visits. Involving the parent or caregiver in the administration of the evaluation has the potential to make any change in scores more obvious over time. It is also a possibility that caregivers may not feel comfortable asking their child to perform tasks that are difficult leading to potential variability in clinic versus home results. As with any study, the parent or caregiver should not be provided with specific testing scores and be reminded that study findings should not be shared with others or posted on social media.

Of particular relevance to the United States, COA are used to monitor progress after initiation of a disease-modifying treatment and are required for ongoing insurance coverage. In this case, the insurance company often requires the use of specific COA. It is possible that the pre-specified COA is not possible or safe to conduct remotely for many reasons. Solutions such as temporary use of an assessment feasible within the home environment may be required and may also permit the PT to add to the narrative of impact of disease-modifying treatment(s).

Another important consideration when remote assessments are performed for a clinical purpose is local licensure/certification laws and guidelines. At the time of publication, most states in the US prohibit telehealth for physical therapy if the PT is not licensed to practice in the state where the patient is located (Bierman et al., 2018). Performing evaluations via telehealth without a license in the state where the patient is located may result in denials for reimbursement for services and/or disciplinary action regarding the therapist’s license. In other regions of the world it is important to determine the legal parameters around which a PT is able to practice. It is the responsibility of the PT to investigate the practice laws for the state or country in which the patient resides.

While licensure is clearly important to ensuring patient safety, there is the need to re-evaluate tele-health restrictions and advocate for policy changes to ensure quality care can be accessed within and outside of a pandemic. In our experience, these licensure restrictions often resulted in lapses of care and/or reduced access to expert care. It is not uncommon that patients must travel out of state to access expert multidisciplinary neuromuscular centres due to lack of experts within their area.

Existing models of PT/CE training in COA to support these studies required adaptation to meet COVID-19 restrictions and ensured the continuation of a high level of training quality and ongoing support for CEs during this challenging time. Prior to the pandemic, PT/CEs who assess patients for research trials were required to undertake extensive training in COAs to ensure highly reliable and valid data acquisition. This training was typically delivered face to face, by a PT Master Trainer, utilizing a combination of didactic teaching, demonstration and practice. With travel heavily restricted across the globe, in person training was no longer possible and we experienced the additional challenge of continuing to provide a high standard of training in COAs remotely. We also encountered an additional challenge where PT/CE staff at many of these centres who were conducting evaluations were deployed to other areas of their hospital or in some centres furloughed, necessitating the training of new PTs and CEs. To maximize training quality and information retention, didactic training sessions were reduced to shorter modules. Videos demonstrating the evaluations were used by the trainer to supplement the didactic training. If patients could visit the clinic during the training, the trainer would view practice sessions in real time and provide feedback. If the PTs first language was not English, simultaneous translation was provided, via a separate line in the online platform. Newly trained PTs were then asked to film a practice videos on a colleague or patient, whoever it was feasible to assess at the time. The trainer would provide written feedback. Consideration needs to be given that not all PTs have received the same tertiary education or have the same scope of work in their region as a PT in another.

Interestingly, while there is often an assumption online training represents an easier, more cost effective alternative to face to face training, we have found the remote training model to be very time and resource intensive, particularly for those brand new CEs who have not been previously trained. While remote training has provided a temporary option, we will quickly revert to our face to face model of training in many regions once this becomes safe and feasible.

## Concluding Remarks

Remote testing and evaluations have the potential to disrupt traditional trial design paradigms and decentralize clinical trials in the future. There are ongoing efforts by our teams, and others within the neuromuscular field, to compare and validate remote evaluations versus in clinic assessments, including a the VCP natural history study (NCT04823143) and Defining Clinical Endpoints in Limb girdle muscular dystrophy (GRASP Defining Endpoints: NCT03981289). As described above, significant effort went in to selecting assessments that could potentially be completed validly and safely within a home environment. However, it is important to understand any differences between these environments and their comparability to inform future trial design. Whilst some commonly used outcome measures in NMD adapted well to the home environment, those involving hands on assessment of a skilled PT could not be delivered successfully via remote testing.

We have discussed the urgent need to modify COA, clinical care and the training of PTs during the COVID-19 global pandemic. We have outlined how COA for patients with NMD were modified to allow for their remote evaluation and how a predominantly face to face training model was tailored for remote delivery. We have discussed the positives as well as the challenges encountered and overcome along the way. Considering it may be sometime until we return to a pre-pandemic model of clinical care and evaluation, we have demonstrated an alternative model that facilitates the continuation of an acceptable standard of clinical care and evaluation for patients with neuromuscular disorders in the clinic and for clinical trials.
